# HTLV-1 infection in bone marrow mesenchymal stem cells isolated from HTLV-1 individuals

**DOI:** 10.1186/1742-4690-11-S1-P106

**Published:** 2014-01-07

**Authors:** Evandra Strazza Rodrigues, Maria do Carmo Favarin, Mayra Dorigan de Macedo, Kátia Kaori Otaguiri, Maristela Delgado Orellana, Osvaldo Massaiti Takayanagui, Patrícia Vianna Bonini Palma, Gil Cunha de Santis, Dimas Tadeu Covas, Simone Kashima

**Affiliations:** 1 Regional Blood Center of Ribeirão Preto, Faculty of Medicine of Ribeirão Preto, University of São Paulo (USP), Ribeirão Preto, Brazil; 2 Faculty of Pharmaceutical Sciences of Ribeirão Preto, USP, Ribeirão Preto, Brazil; 3 Department of Tropical Medicine, Faculty of Medicine of Ribeirão Preto, USP, Ribeirão Preto, Brazil; 4 Department of Clinical Medicine, Faculty of Medicine of Ribeirão Preto, USP, Ribeirão Preto, Brazil

## 

The characterization of cell types that are susceptible to HTLV-1 infection is essential to understand of the biology of virus infection and the pathophysiology of HTLV-related diseases. In the present study, we investigated bone marrow (BM) cells from asymptomatic carriers (HAC) and symptomatic HTLV-1 individuals. Initially, we observed an infiltrated of CD4^+^ T-cell lymphocytes in BM from HTLV-1 individuals when compared to healthy controls (p≤0.02). The proviral DNA of BM HTLV-1 CD4^+^ T cells revealed the presence of integrated provirus. The number of fibroblast progenitor cells, referred as colony-forming units-fibroblasts (CFU-F), were lower in HTLV-1 infected individuals (CFU-F per 5 x 10^5^ cells in HAM/TSP (2.1±1.6), HAC (7.5±2.1) compared to healthy controls (10.4±1.1). HTLV-1 BM mesenchymal stem cells (MSC) isolated showed surface expression of CD105, CD73, and CD90, absence of hematopoietic markers such as CD45, CD34, and CD14; and in vitro differentiation to adipogenic and osteogenic cells. Proviral HTLV-1 DNA was detected in MSC from all HTLV-1 patients and the levels ranged from 2.5 to 25.7 copies per 10^5^ cells. HTLV-1 MSC was also positive to HTLV-1 p19 protein as determined by confocal microscopy. The confirmation of active viral replication was performed in the concentrated supernatant obtained from MSC by real-time PCR of the pol and gag HTLV-1 gene. In conclusion, we suggest that HTLV-1 could infect and replicate in human MSC possibly by the contact with infected CD4^+^ T-cells.

## Financial support

FUNDHERP, CTC/FAPESP, INCTC, and CNPq.

